# Imputation of Human Primary Osteoblast Single Cell RNA-Seq Data Identified Three Novel Osteoblastic Subtypes

**DOI:** 10.31083/j.fbl2710295

**Published:** 2022-10-31

**Authors:** Hui-Xi Zhang, Chong Cao, Xiao-Hua Li, Yan Chen, Yue Zhang, Ying Liu, Yun Gong, Xiang Qiu, Cui Zhou, Yu Chen, Zun Wang, Jun-Xiao Yang, Liang Cheng, Xiang-Ding Chen, Hui Shen, Hong-Mei Xiao, Li-Jun Tan, Hong-Wen Deng

**Affiliations:** 1 Laboratory of Molecular and Statistical Genetics, College of Life Sciences, Hunan Normal University, 410081 Changsha, Hunan, China; 2 Tulane Center of Biomedical Informatics and Genomics, Deming Department of Medicine, Tulane University School of Medicine, New Orleans, LA 70112, USA; 3 School of Basic Medical Science, Central South University, 410008 Changsha, Hunan, China; 4 Xiangya Nursing School, Central South University, 410013 Changsha, Hunan, China; 5 Department of Orthopedics, Xiangya Hospital, Central South University, 410008 Changsha, Hunan, China; 6 Department of Orthopedics and National Clinical Research Center for Geriatric Disorders, Xiangya Hospital, Central South University, 410008 Changsha, Hunan, China; 7 Center of Reproductive Health, System Biology and Data Information, Institute of Reproductive & Stem Cell Engineering, School of Basic Medical Science, Central South University, 410081 Changsha, Hunan, China

**Keywords:** single-cell RNA sequencing (scRNA-seq), imputation, osteoblast heterogeneity, immune regulation, osteoclast differentiation, adipose differentiation

## Abstract

**Background:**

Recently, single-cell RNA sequencing (scRNA-seq) technology was increasingly used to study transcriptomics at a single-cell resolution, scRNA-seq analysis was complicated by the “dropout”, where the data only captures a small fraction of the transcriptome. This phenomenon can lead to the fact that the actual expressed transcript may not be detected. We previously performed osteoblast subtypes classification and dissection on freshly isolated human osteoblasts.

**Materials and Methods:**

Here, we used the scImpute method to impute the missing values of dropout genes from a scRNA-seq dataset generated on freshly isolated human osteoblasts.

**Results:**

Based on the imputed gene expression patterns, we discovered three new osteoblast subtypes. Specifically, these newfound osteoblast subtypes are osteoblast progenitors, and two undetermined osteoblasts. Osteoblast progenitors showed significantly high expression of proliferation related genes (*FOS, JUN, JUNB* and *JUND*). Analysis of each subtype showed that in addition to bone formation, these undetermined osteoblasts may involve osteoclast and adipocyte differentiation and have the potential function of regulate immune activation.

**Conclusions:**

Our findings provided a new perspective for studying the osteoblast heterogeneity and potential biological functions of these freshly isolated human osteoblasts at the single-cell level, which provides further insight into osteoblasts subtypes under various (pathological) physiological conditions.

## 1. Introduction

Osteoblasts are bone forming cells, which account for 4% to 6% of the cellular content within the bone lineage [1]. Osteoblasts were differentiated from bone marrow derived skeletal stem cells (SSCs) [2,3], *Runx2* and osterix (*Sp7*) are two critical transcription factors to regulate the differentiation of osteoblast [4,5]. Osteoblasts cover the active bone surface, and their main function is to produce new bones through the synthesis and assembly of extracellular matrix. Although osteoblasts are mainly involved in bone modeling and remodeling, previous studies have shown that osteoblasts can also regulate immune cells and inflammation. Specifically, osteoblasts can attract B and T lymphocytes, monocytes, and neutrophils to the site of inflammation and take part in immunomodulation through expressing interleukin 6 (IL-6), monocyte chemoattractant protein-1 (MCP-1/CCL2), and CXC Chemokine Ligand 2 (CXCL2) [6–10]. Osteoblasts and adipocytes can regulate each other through expressing some specific genes [11,12]. The imbalance of the ratio of adipocytes to osteoblasts in the bone marrow may be an important factor leading to osteoporosis [13,14].

Cellular heterogeneity is an essential feature of different cell groups. The subpopulations of cells that cause cell heterogeneity can be determined by differences in gene expression profiles [15]. In bulk RNA-seq, cellular heterogeneity cannot be completely addressed since signals of differentially expressed genes would be averaged across cells. However, single-cell RNA sequencing (scRNA-seq) technology is now becoming a powerful tool to capture whole cell transcripts at the single-cell level. scRNA-seq can quantify the heterogeneity within the population at single-cell resolution, which may reveal heterogeneous rare cell populations in complex tissues or classical types of cells [16–19]. In recent years, some studies have applied scRNA-seq to mouse osteoblasts. For example, one study identified subtypes of preosteoblasts and mature osteoblasts based on the osteoblast transcription profiles [20], while another study divided osteoblasts into three subgroups (undetermined osteoblasts, osteogenic transdifferentiated osteoblasts and mature osteoblasts) [21]. Recently, we performed the first scRNA-seq study on freshly isolated human osteoblasts and identified three different osteoblast subtypes and their differentiation relationships [22]. We found that different subgroups have different functional characteristics in the regulation of bone metabolism and angiogenesis [22].

However, an important characteristic of scRNA-seq data is the phenomenon of “dropout”, in which a gene is observed at a medium expression level in one cell, but undetected in another cell [23,24]. Generally speaking, these events occur due to the low mRNA content in some individual single cells [24]. The low starting amount makes some mRNAs completely lost during reverse transcription and cDNA amplification, which then cannot be detected in the subsequent sequencing [22]. Therefore, the actual expressed transcript may not be detected when sequencing in some cells, which may bias downstream analysis [25,26]. To tackle this problem, several imputation methods have been proposed [27–29]. Bulk RNA-seq measures the average gene expression. scRNA-seq can detect gene expression at single-cell resolution [22,24]. The data fluctuation of scRNA-seq was hence larger than that of bulk RNA-seq. Therefore, some imputation methods are only applicable to bulk RNA sequences, and may not be directly applicable to scRNA-seq data [28,30,31]. The imputation method that was more suitable for scRNA-seq, for example, the imputation methods MAGIC [32] and SAVER [33] may change the expression level of all genes, including those unaffected by dropouts, which will introduce new bias. Recently, Li *et al.* [23] developed a novel statistical method, called scImpute, for accurate and reliable imputation of the dropouts of scRNA-seq data. scImpute can automatically identify likely dropouts by fitting a mixture model for each cell type, and only perform imputation on these values, without introducing new deviations to the rest data [23]. It can also detect outlier cells by using the most similar linear regression model and exclude them from imputation. Also, scImpute has a good effect in clustering, detecting differentially expressed genes (DEGs) and improving the reconstruction of subsequent cell development trajectories [24].

In this study, we used the scImpute method to impute the scRNA-seq data of freshly isolated human osteoblasts. After the imputation and recalculation, we obtained more osteoblast clusters compared with the original data. We then determined the different functional characteristics of each novel osteoblast subtypes in terms of bone homeostasis, the differentiation of adipocytes and osteoclasts, immune regulation, osteoblast proliferation and regulation of extracellular matrix production, which may provide a better understanding about the heterogeneity and functions of osteoblasts.

## 2. Materials and Methods

### 2.1 scRNA-Seq Data

The scRNA-seq data was generated from the femoral head-derived osteoblasts of a subject with osteoarthritis and osteopenia (GSE147390), which has been described in detail in our previous publication [22]. In brief, we used fluorescence-activated cell sorting (FACS) to collect ALPL^+^/CD45/CD34/CD31^−^ cells [34] as osteoblasts from the femoral head bone tissue sample. scRNA-seq libraries were prepared using Single Cell 3’ Library Gel Bead Kit V3 following the manufacturer’s guidelines (https://support.10xgenomics.com/single-cell-gene-expression/library-prep/doc/user-guide-chromium-single-cell-3-reagent-kits-user-guide-v3-chemistry) [22]. After obtained the raw sequencing data, we used Cell ranger3.0 to demultiplex and map cell barcodes to the human transcriptome (GRCh38/hg38) (https://support.10xgenomics.com/single-cell-gene-expression/software/pipelines/latest/what-%20is-cell-ranger). Create Cell Ranger-compatible reference genomes according to the instructions of 10x Genomics (http://www.10xgenomics.com), and finally generate a digital gene expression matrix [22].

### 2.2 Imputing Dropout and Pre-Processing of scRNA-Seq Data

In order to deal with the dropout event, we used the R package scImpute v0.0.9 (Los Angeles, CA, USA) [23] and performed imputation calculations on 9801 cells using default parameters as input. Basic algorithm of scImpute is learns each gene’s dropout probability in each cell by fitting a mixture model for each cell type. Then, scImpute imputes the dropout values of genes in a cell by borrowing information of the same gene in other similar cells [23,24].

For further quality control, we removed cells with less than 150 detected genes. After that, we calculated the distribution of genes detected in each cell, and removed any cells in the top 2% quantile, as well as the cells whose transcription volume >20% was attributed to mitochondrial genes. The gene counting matrix was converted into a Seurat object by Seurat R package [22,35]. We used the Normalize-Data function in the Seurat R software package to normalize the screened gene expression matrix. The number of UMIs for each gene was divided by the total number of UMIs for each cell and multiplied by 10,000 and then the result was log transformed [22].

### 2.3 Dimensionality Reduction and Data Visualization

In order to visualization the data, we projected the standardized gene expression matrix onto a two-dimensional panel. We selected the top 2000 genes with the largest variation for principal component analysis (PCA), and reduced the data to the first 19 PCs (according to the standard deviation of the principal components, corresponding to the platform area of the “elbow diagram”) for unified manifold approximation and projection (UMAP) dimensionality reduction [22,36]. After data visualization, we applied the clustering method based on unbiased graphs for clustering analysis [37]. For DEGs analysis, we used the Wilcoxon Rank-Sum test to find genes that exhibited significantly higher expression (false discovery rate (FDR) <0.05) in a specific cluster compared to other clusters [22].

### 2.4 Pathway Enrichment Analysis and Trajectory Inference Analysis

We used the clusterProfiler to perform gene ontology (GO) and Kyoto encyclopedia of genes and genomes (KEGG) pathway analyses to enrich the significant terms and pathways on each novel osteoblast subtypes [38]. Then we used the Diffusion-Maps function in the destiny (v3.0.0) to reconstruct the development trajectory of a single unit in pseudotime order [39,40]. The principle of this analysis was to reorder the asynchronously differentiated cells according to their potential development condition and classify the cells along their developmental trajectories.

## 3. Result

### 3.1 Osteoblasts Identification

**Fig. 1.**
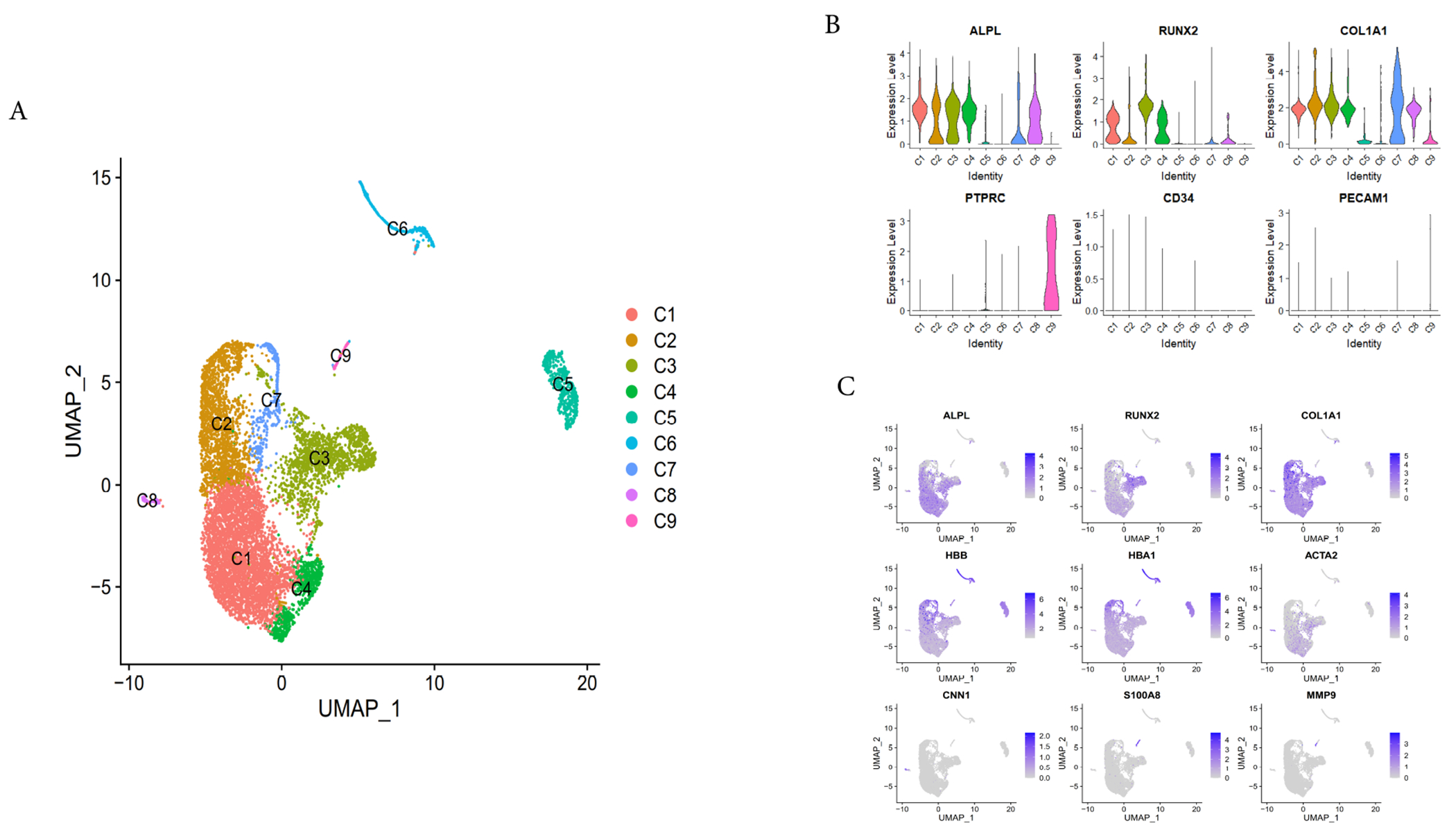
Osteoblasts isolation and identification. (A) UMAP dimension reduction of isolated cells, colored by different clusters. (B) Osteoblasts were selected using known cell markers. ALPL, Runx2 and COL1A1 are specific markers of osteoblasts, while PTPRC (CD45), CD34 and PECAM1 (CD31) are markers of endothelial and hematopoietic cells. (C) The known cell markers of each subpopulation were expressed on the umap dimension reduction map. The first three markers in the first row (ALPL, Runx2, and COL1A1) were osteogenic markers. HBB and HbA1 are markers of nucleated red blood cell clusters C5 and C6. The last four genes are markers of C8 (smooth muscle cell clusters) and C9 (neutrophil clusters).

The scRNA-seq data used in this study have been detailed described in our previous publication [22]. It contains 9801 cells. We used the scImpute method to calculate the dropout rate of osteoblast scRNA-seq data and performed imputation only on those missing values. We obtained 9425 cells, with an average of 6659 genes detected in each cell after the QC (quality control). However, after quality control of the data without imputation, only 8557 cells were obtained, and only 2365 genes were detected on average per cell [22]. The quality control standards we used here were consistent with our previous analysis [22]. We used Unified Manifold Approximation and Projection (UMAP) [36], to project high dimensional gene expression profiles onto two-dimensional panels to visualize cellular heterogeneity (Fig. 1A). When the clustering was completed, we obtain nine distinct cell subsets (C1-C9) by the k-nearest neighbor algorithm [35], and used Wilcoxon test to determine their cluster-specific marker genes (Supplementary Fig. 1). Consistent with our previous study [22], we excluded several contaminant cell types, including two erythrocyte clusters (C5 and C6) which had a high expression levels of HBB and HBA1, a smooth muscle cell cluster (C8) with a high expression levels of *ACTA2* and *CNN1*, a neutrophil cluster (C9) with a high expression levels of *S100A8* and *MMP9* [22], so we focused our subsequent analysis on clusters C1, C2, C3, C4 and C7, which show high expression of osteoblast-specific markers (i.e., *ALPL, RUNX2* and type 1 collagen (*COL1A1*)), although C7 had a high expression levels of osteoblast-specific markers, but it also showed a significantly high expression levels of mitochondrial genes, suggesting that C7 was undergoing apoptosis, so C7 was excluded from the subsequent analysis (Supplementary Fig. 1).

### 3.2 Transcriptional Profiling of Human Osteoblasts

**Fig. 2.**
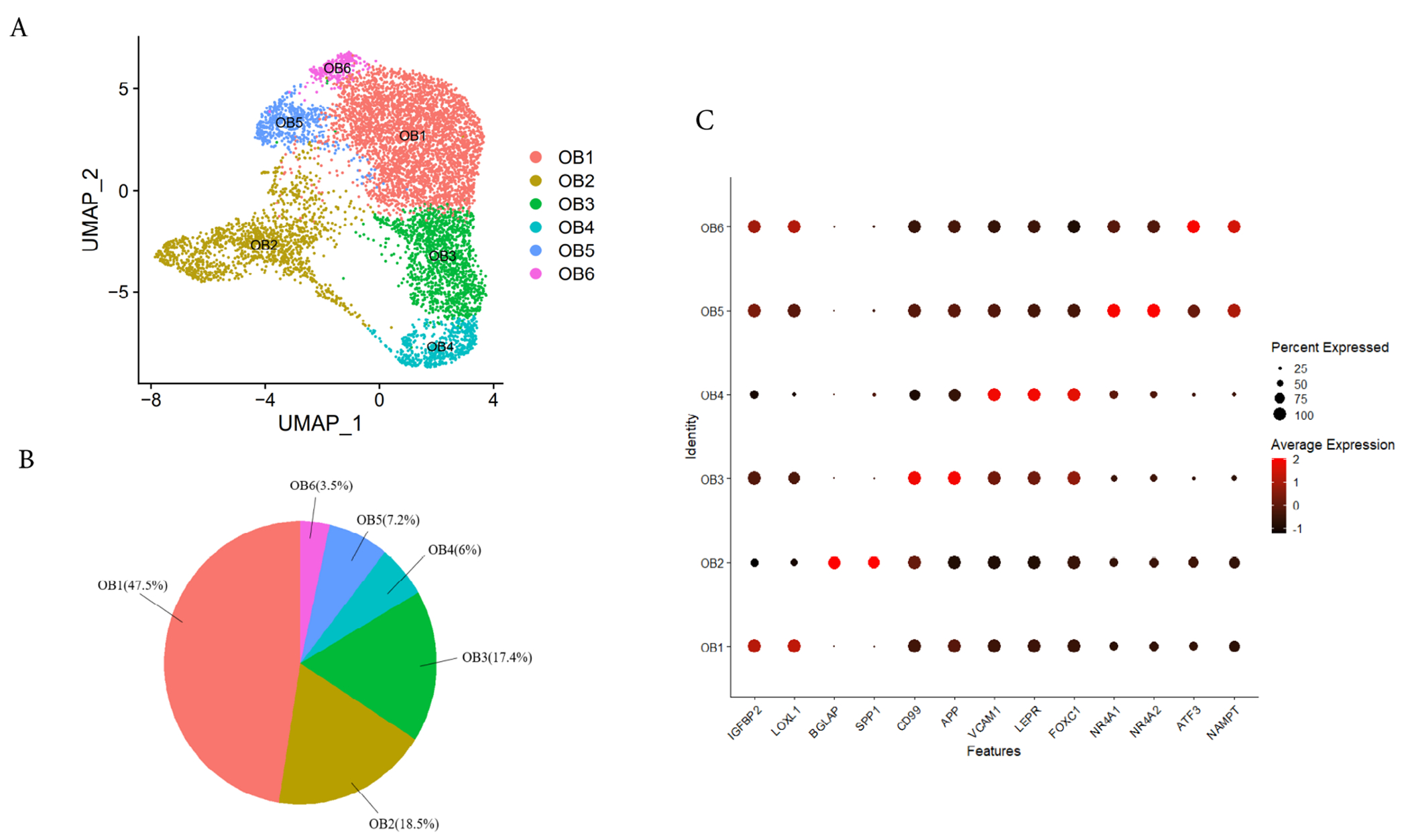
scRNA- seq analysis of human osteoblasts. (A) Six osteoblast clusters. The UMAP (Unified Manifold Approximation and Projection) of 7656 osteoblasts was shown by cluster staining. (B) Proportion of six osteoblast clusters. Colored by clustering. (C) Cluster characteristic genes. The dot plot showed the logarithmic transformation normalized expression levels of the marker genes with highest expression for each cluster OB1, OB2, OB3, OB4, OB5 and OB6.

To further study the heterogeneity within osteoblasts, we selected clusters C1, C2, C3 and C4 with high expression of *ALPL, RUNX2* and *COL1A1* (Fig. 1B). Here, we performed the second round of data quality control, removing the cells with >5% of the transcripts attributed to mitochondrial genes [22], and obtained 7656 cells for further analysis, after the reclustering, we identified six osteoblast subtypes (Fig. 2A,B,C), which were labeled: (1) Osteoblasts 1 (OB1, 48.20%), expressing high levels of insulin-like growth factor binding protein 2 (*IGFBP2*) and lysyl oxidase like 1 (*LOXL1*); (2) Osteoblasts 2 (OB2, 18.39%), highly expressing osteoblasts maturation markers (bone gamma carboxyglutamic acid protein (also known as osteocalcin, *BGLAP*), secretory phosphoprotein-1 (also known as osteopontin, *SPP1*), integrin binding sialoprotein (*IBSP*)) and two osteoblasts differentiation transcription factors *RUNX2* and *SP7* [20,41,42]; (3) Osteoblasts 3 (OB3, 16.85%), not only expressing MSC specific markers (e.g., leptin receptor (*LEPR*). vascular cell adhesion molecule 1 (*VCAM1*) [22,43,44] but also distinctively expressing high levels of *CD99* and amyloid beta precursor protein (*APP*). (4) Osteoblast 4 (OB4, 7.22%), significantly expressed high levels of *LEPR* [44,45] and Forkhead box C1 (*FOXC1*), so it could be a osteoblast progenitors [46]; (5) Osteoblasts 5 (OB5, 6.06%), significantly expressing high levels of nuclear receptor subfamily 4 members 1 and 2 (*NR4A1* and *NR4A2*); (6) Osteoblasts 6 (OB6, 3.28%), significantly expressing high levels of activated transcription factor 3 (*ATF3*) and nicotinamide phosphoribosyl transferase (*NAMPT*), considering that the gene expression patterns of OB1, OB5 and OB6 were different from those of clusters OB2, OB3 and OB4, so we defined OB1, OB5 and OB6 as undetermined osteoblasts. Notably, the osteocytes marker genes (*SOST1* and *DMP1*) can hardly be detected in this imputed transcriptomic data, suggested that the protocol of osteoblast isolation were not suitable for osteocyte isolation.

We found that after imputation, apart from the three osteoblast subtypes found in the previous analysis (preosteoblasts (OB3), mature osteoblasts (OB2) and undetermined osteoblasts (OB5, *NR4A1^high^/NR4A2^high^*)) [22], we discovered three new unique cell subgroups, including OB4 (*LEPR^high^/FOXC1^high^*) and OB1 (*IGFBP2^high^/LOXL1^high^*) and OB6 (*ATF3^high^/NAMPT^high^*). At the same time, the imputed data increased the number of osteoblasts for analyses from 5329 to 7656, an increase of about 24% (Supplementary Fig. 2 A,B) scImpute effectively imputed the missing values in the scRNA-seq data of osteoblasts. In the original data without imputation, an average of 2365 genes were detected per cell, but after the imputation, an average of 6659 genes were detected per cell (Supplementary Fig. 2C).

### 3.3 Dynamic Gene Expression Patterns in Different Developmental Stages of Osteoblasts

**Fig. 3.**
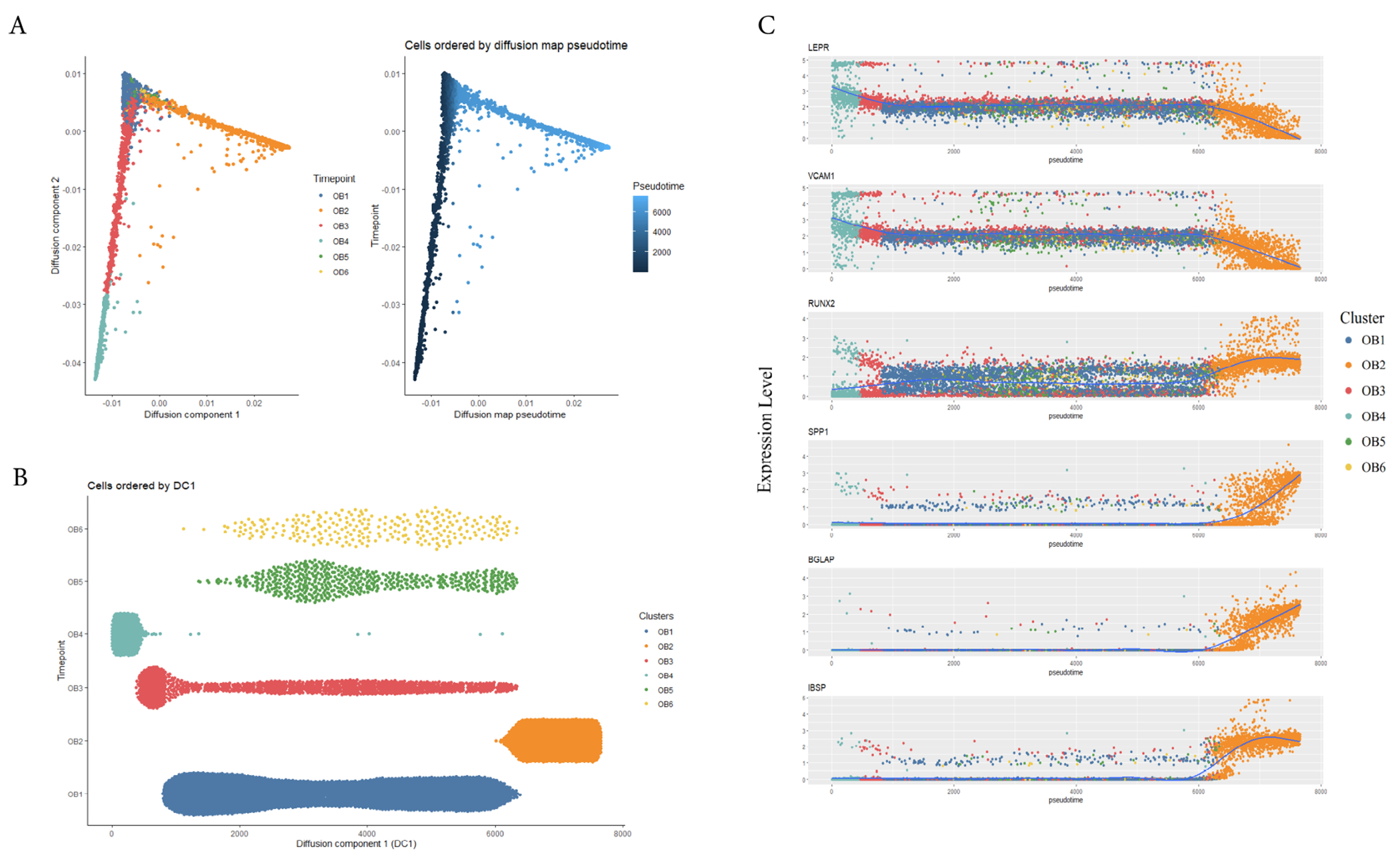
Trajectory Inference of Human Osteoblasts. (A) Reconstructed cell differentiation trajectory of human osteoblasts, colored by subpopulation identities. The right trajectory plot in the square indicated the direction of pseudotime. (B) Distribution of each cell subpopulation along the pseudotime. (C) Expression levels of indicated genes in the six osteoblast subtypes with respect to their pseudotime coordinates. The x-axis indicates the pseudotime, while the y-axis represents the gene expression levels. The color corresponds to the six different osteoblast subsets. Blue lines depict the LOESS regression fit of the normalized expression values.

We used diffusion maps to reconstruct the development trajectories of the six identified osteoblast clusters [40]. This analysis revealed the differentiation process of osteoblasts. In our reconstructed lineage branch, all cells were remained in one cell lineage trajectory (Fig. 3A). We found that osteoblast progenitors (OB4) were mainly enriched in the initial stage of pseudotime, and preosteoblasts (OB3) were distributed in the early stage of pseudotime trajectory, undetermined osteoblasts (OB1, OB5 and OB6) were concentrated in the middle stage of pseudotime, and mature osteoblasts (OB2) were mainly enriched in the terminal stages of the osteoblastic lineage trajectory (Fig. 3A,B). To support trajectory inference, we further analyzed the transcriptional continuum of the cell lineage. By comparing the MSCs and osteoblasts specific marker genes expression patterns during the pseudotime trajectory, we found that the expression of MSCs markers (e.g., *LEPR* and *VCAM1*) decreased with the prolongation of pseudotime and osteoblast markers (e.g., *RUNX2, BGL4P, SPP1* and *IBSP*) were highest in the final stage of pseudotime. This result was consistent with the results of other studies [22,47]. Interestingly, the undetermined osteoblasts (OB1, OB5 and OB6) were mainly concentrated in the middle stage of the pseudotime (Fig. 3C), which suggested that they may be three subtypes of osteoblasts with different functions in the middle stage.

### 3.4 Proliferation Function of Osteoblast Progenitors

**Fig. 4.**
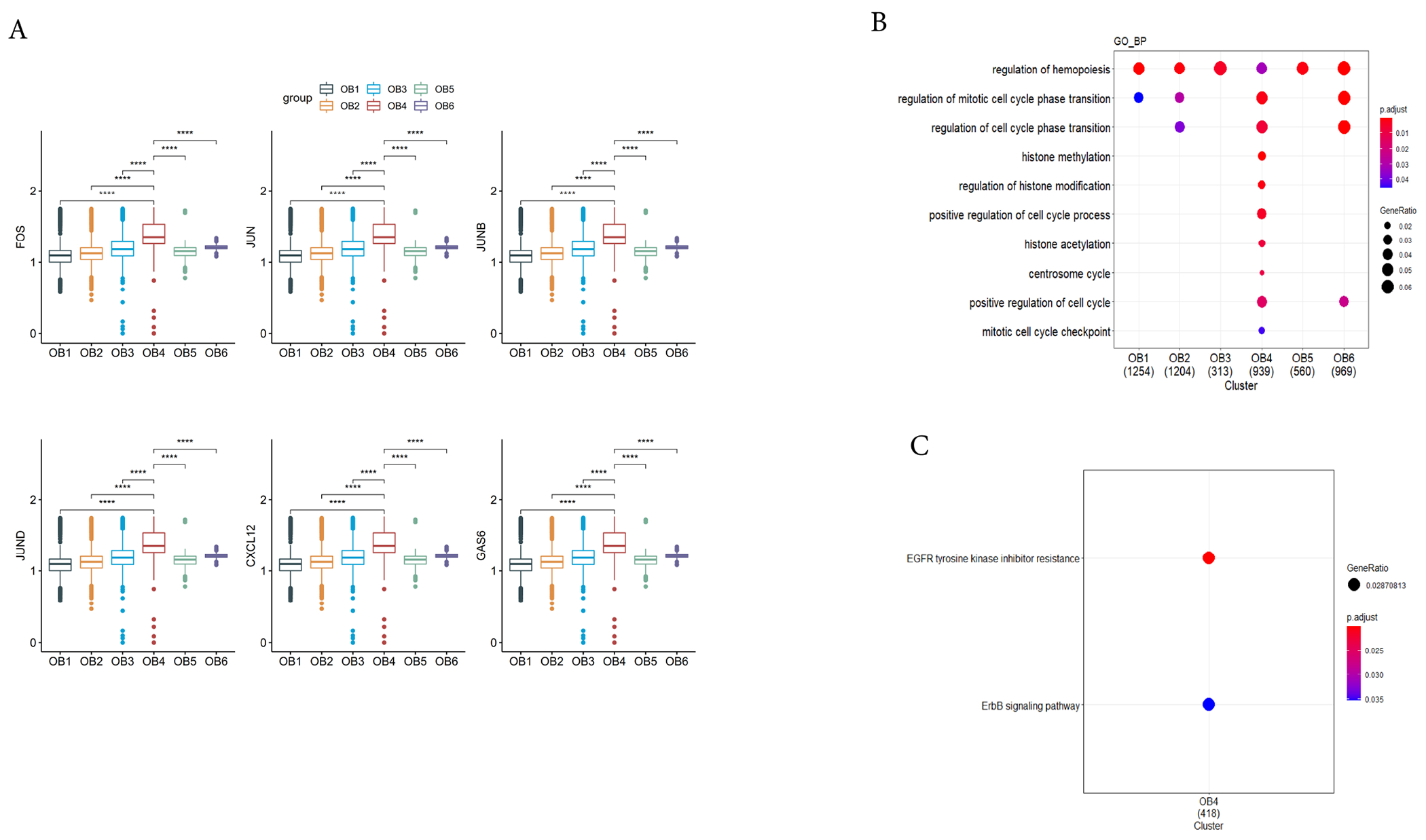
Proliferation of osteoblast progenitors. (A) Cell proliferation related genes enriched in cluster OB4. X-axis represents the cluster and y-axis reflects log-normalized gene expression levels. The data are mean ± standard deviation. Stars indicate the significance levels of the gene expression difference between two clusters by Wilcoxon signed-rank test. N.S., not significant, *p-adjusted ≤ 0.05, **p-adjusted ≤ 0.01, ***p-adjusted ≤ 0.005, ****p-adjusted ≤ 0.001. (B) Cell proliferation related GO (Gene Ontology) term enriched in cluster OB4. The x-axis represents the clusters and the y-axis represents the GO terms related to the cell proliferation regulation. The size of the dots indicates the gene ratio and the color indicates the adjusted *p*-values. (C) Cell proliferation related KEGG (Kyoto Encyclopedia of Genes and Genomes) terms enriched in cluster OB4.

It is known that the developmental stages of osteoblasts have three main periods: the proliferation period, the extracellular matrix production period, and the extracellular matrix mineralization period [48]. Osteoblast progenitors (OB4) are in the cell proliferation stage. Compared with the other clusters, osteoblast progenitors (OB4) showed significantly high expression of *FOS, JUN, JUNB* and *JUND* (Fig. 4A). Studies have shown that the main feature of the stage was the production of histones, FOS, FOSB, JUN, and p21, *etc*. [48–50]. They were highly expressed in the proliferation stage, but their expression declined rapidly after proliferation [51–54]. Knocking out *FOS* can inhibit the proliferation of osteoblasts [55]. Next, we used DEGs in osteoblast progenitor cells for GO enrichment analysis. We noticed that the terms related to cell proliferation were enriched, including “regulation of cell cycle phase transition”, “histone modification”, *etc*. (Fig. 4B). Kyoto Encyclopedia of Genes and Genomes (KEGG) pathway analysis showed that two metabolic pathways were highly enriched, including “EGFR tyrosine kinase inhibitor resistance” and “ErbB signaling pathway” (Fig. 4C). Studies have shown that the lack of EGFR can reduce the proliferation of osteoblast progenitors (OB4), and EGFR promoted the proliferation of osteoblasts by activating the phosphorylation of ERK1/2 of the downstream signal transduction ERK pathway [56,57]. Studies have reported that osteoblasts proliferation maintenance was related to ErbB family signaling of receptor tyrosine kinases [58]. In addition, GO enrichment analysis also showed enrichment of the functional pathway of “regulation of hematopoiesis”, which is a known function of osteoblasts. At the same time, osteoblast progenitors (OB4) highly expressed *CXCL12* and *GAS6* (Fig. 4A). CXCL12 was known to play an important role in maintaining HSC homeostasis and hematopoiesis [20]. GAS6 positively regulated CD34+ hematopoietic progenitor cells (HPCs) [59].

### 3.5 Two Osteoblast Subpopulations Involved in Osteoclastogenesis and Adipogenesis

**Fig. 5.**
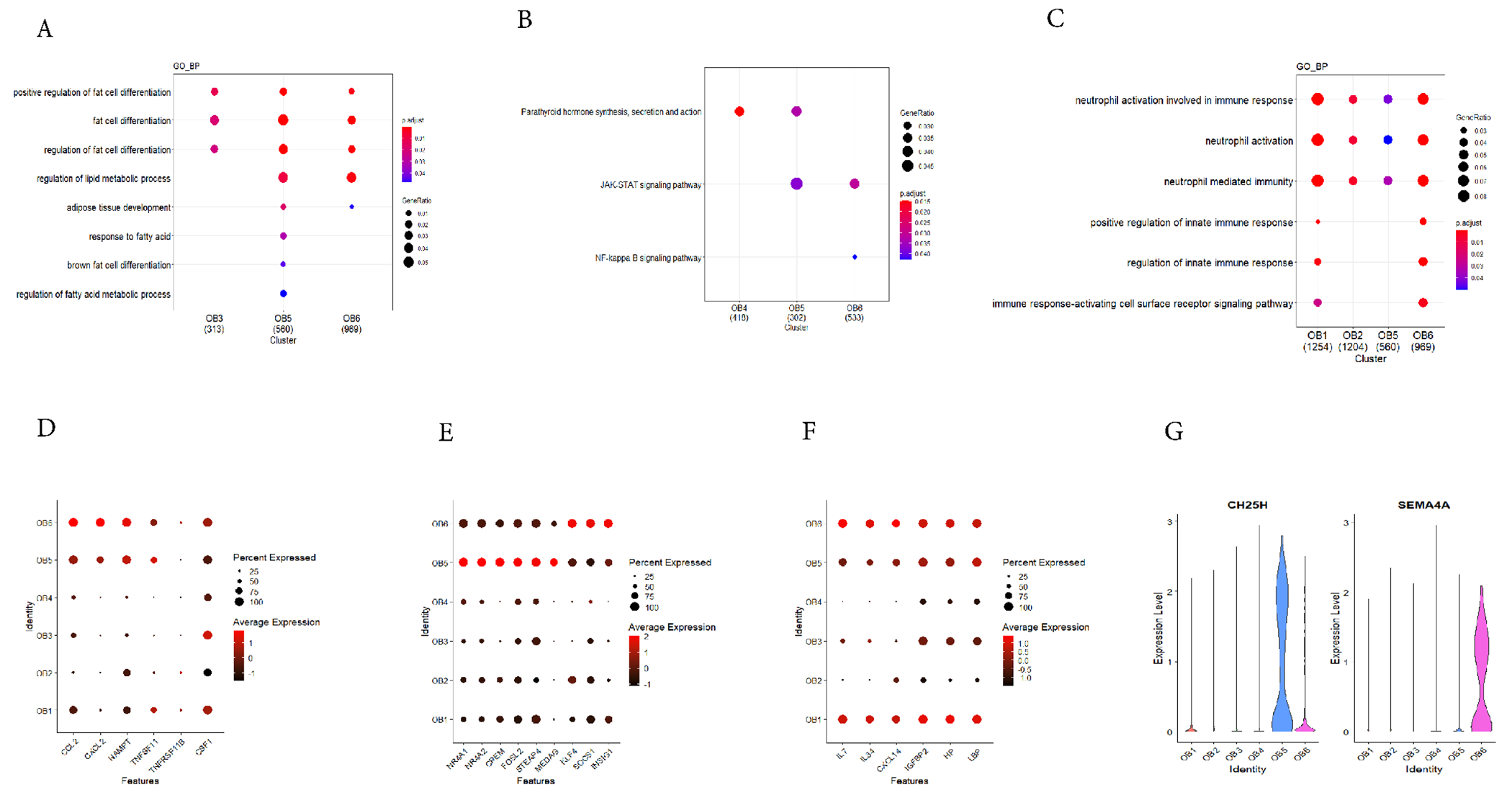
Osteoblast population regulates adipocyte differentiation. (A) Regulation of adipogenesis related GO terms enriched in clusters OB5 and OB6. (B) Osteoclasts development related KEGG terms enriched in clusters OB5 and OB6. (C) Immune regulation related GO terms enriched in OB clusters. (D) Regulation of osteoclast differentiation related genes enriched in OB clusters. (E) Regulation of adipogenesis related genes enriched in OB clusters. (F) The genes involved in immune regulation enriched in OB clusters. (G) The expression levels of OB5 and OB6 specific surface marker genes in the OB clusters.

There was evidence that osteoblasts can regulate the production of osteoclasts [60–62]. In our previous article [22], it has been mentioned that the high expression of *NR4A1* and *NR4A2* in the subpopulation of undetermined osteoblasts (OB5) can inhibit the formation of osteoclasts. Interestingly, we found that OB6 may possibly promote the formation of osteoclasts compared with other osteoblast groups. Some cytokine produced by osteoblast, such as *CCL2, CXCL2, NAMPT* and *TNFSF11* (RANKL) were highly expressed in OB6 (Fig. 5D). *TNFSF11* has been proved to be a crucial gene in the process of the osteoclast development [63], We found that TNFSF11 was highly expressed in OB5 and OB6. CCL2 could be regulated by parathyroid hormone (iPTH) to promote osteoclast formation [64]. CXCL2 can be activated by NF-kappaB ligand receptors through the JNK and NF-kappaB signaling pathways in osteoclast precursor cells, thereby promoting the production of osteoclasts [65]. NAMPT secreted by osteoblasts promoted osteoclast recruitment by increasing the production of RANKL [66]. The KEGG pathway analysis showed that osteoclast development related terms of “NF-kappa B signaling pathway”, “JAK-STAT signaling pathway” and “Parathyroid hormone synthesis, secretion and action” were enriched in OB5 and OB6 (Fig. 5B).

Studies have reported that osteoblasts and adipocytes can secrete some cytokines to regulate each other’s differentiation, and under certain conditions, osteoblasts could express some adipocyte-specific genes [11,12]. We found that OB5 not only had the possible function of regulating osteoclasts, but may also participated in adipogenesis. After extracting the high expression genes and performing GO pathway analysis, we found that GO terms “regulation of fat cell differentiation” and “regulation of lipid metabolic process” were mainly abundant in OB5 and OB6, but not in other subpopulations (Fig. 5A). In order to determine the special role of the two osteoblast subpopulations in adipogenesis, we further checked the expression patterns of genes related to the regulation of adipogenesis in the two clusters.

*NR4A1* and *NR4A2*, which were highly expressed in OB5, acted as inhibitors for adipogenesis in adipocytes and were induced by cAMP signal. We also found that cAMP-responsive element modulator (*CREM*) was highly expressed in OB5 (Fig. 5E) [67–69]. Some adipocyte function related genes were also highly expressed in OB5 and OB6 (Fig. 5E), FOSL2 inhibited the differentiation of adipocytes by controlling the expression of adiponectin [70]. MEDAG can regulate the differentiation of preadipocyte and accumulate lipid [71]. STEAP4 enhanced the insulin-stimulated glucose uptake in adipocyte. *KLF4, SOCS1* and *INSIG1* were highly expressed in OB6 (Fig. 5E). Studies have shown that KLF4 was essential for adipocytes production *in vitro* and can promote adipocyte production [72]. SOCS1 regulated the differentiation of preadipocytes through C/EBP*α* and PPAR*γ*. The high expression of SOCS1 can promote the formation of adipocytes [73]. INSIG1 could regulate the storage of fat in adipocyte [74]. Since the pseudotime trajectory analysis showed the differentiation level of OB5 and OB6 were overlapped with preosteoblast (OB3), previous studies showed that preosteoblast could form lipid droplets under pathological and aging conditions [75] and preosteoblast cell line MC3T3-E1 cells can undergo adipocytic transdifferentiation under the control of estrogen by canonical Wnt signaling pathway [76]. We speculated OB5 and OB6 possibly were the intermediate osteoblasts with adipogenic properties.

The above results suggested that the OB5 and OB6 might possibly affect the development of osteoclasts and adipogenesis by expressing specific genes, OB5 and OB6 may play important roles in balancing the relative proportions of osteoclasts and participating in adipogenesis *in vivo*. Besides, two specific surface marker genes (*CH25H, SEMA4A*) were detected from OB5 and OB6, which meant these cells may be specifically isolated by Fluorescence activated Cell Sorting (FACS) for subsequent experimental analysis (Fig. 5G). The results of this study may thus provide novel ideas for future treatment/prevention of osteoporosis and obesity by increasing the number or functions of the OB5.

### 3.6 Immunomodulatory Ability of Osteoblast Subpopulations

We further found that in addition to the cytokines CXCL12, CCL2 and NAMPT, there were more immune-related cytokines like IL7, IL34 and CXCL14 highly expressed in OB6 and OB1 (Fig. 5F). Studies have reported that IL7 derived from osteoblast can promote development of lymphocytes in immune response during inflammation [77]. IL34 had a similar function to CSF1 (M-CSF) and could induce the differentiation of osteoclast and increase the number of CD11b^+^ cells [78]. CCL2 was also a known therapeutic target for inflammatory bone destruction diseases. It can control the migration of monocytes and macrophages during inflammation, regulate the positioning and transport of immune cells to participate in immunomodulation [7,79]. CXCL2 and CXCL14 can regulate immune response by controlling the immune cells migration [65,80]. NAMPT secreted by osteoblasts participated in the inflammatory response of osteoarthritis by promoting the release of IL6 and the expression of monocyte chemoattractant protein 1 by osteoblasts [81].

Three secrete protein genes *IGFBP2*, haptoglobin (*HP*) and lipopolysaccharide binding protein (*LBP*) were highly expressed in OB1 (Fig. 5F). Studies have reported that IGFBP2 was involved in immunosuppressive activity [82]. HP participated in immunomodulation by affecting the activity of immune cells (such as T cells, macrophages, *etc*.) [83]. LBP interacted with lipopolysaccharide (LPS) and CD14 to participate in immunomodulation response [84].

We further performed GO enrichment analysis and found that the OB1 and OB6 were enriched in terms related to immunomodulation, such as “neutrophil mediated immunity”, “neutrophil activation involved in immune response”, “positive regulation of innate immune response”, and “regulation of innate immune response” *etc*. (Fig. 5C). These results indicated that the OB1 and OB6 may be important for regulation of the immune system during inflammation. Based on the results above, we speculated that OB1 possibly was the intermediate osteoblasts with immunomodulatory properties, and the OB6 was the intermediate osteoblasts with adipogenesis and immunomodulatory properties. However, further functional experiments are needed to validate the assumptions.

## 4. Discussion

In this study, we used scImpute to calculate the missing values due to the dropout events in the freshly isolated human osteoblasts scRNA-seq data. scImpute focuses on imputing the missing expression values of dropout genes while retaining the expression levels of genes that were largely unaffected by dropout events [23]. The original data after imputation have been greatly improved compared with the previous analysis [22], and the real transcriptome dynamics that have been masked were further restored. We have identified several cell types *in vivo* in humans without any *in vitro* culture from human bones, divided them into nine subgroups. We further revealed that these osteoblasts subtypes might play differential roles in bone formation, osteoclastogenesis, adipogenesis and immunomodulatory based on their unique gene expression patterns, which further illustrated the utility and necessity of imputation of scRNA-seq data.

Here, we focused on some key findings. In addition to those osteoblast subtypes found in the previous analysis [22], including preosteoblasts (OB3), mature osteoblasts (OB2), and undetermined osteoblast (OB5, *NR4A1^high^/NR4A2^high^*), we also found three novel osteoblast subtypes, including known osteoblast progenitors (OB4), a rare osteoblast subtype (OB1) expressing *IGFBP2* and *LOXL1*, and another rare osteoblast subtype (OB6) expressing *ATF3* and *NAMPT*. According to the gene expression pattern and the inferred osteoblast lineage trajectory, we found that: (1) Osteoblast progenitors (OB4, *LEPR^high^/FOXC1^high^*) ranked first in the differentiation lineage, mainly involved in osteoblast proliferation and inducing hematopoiesis; (2) Preosteoblasts (OB3, *CD99^high^/APP^high^*) were located in the early stage of the lineage, and have functions in formation of ECM organization during bone formation processes as well as inducing hematopoiesis as detailed previously [22]; (3) Intermediate osteoblasts (OB1, *IGFBP2^high^/LOXL1^high^*) were in the middle stage of the lineage, and may involve in immunomodulatory function together with OB6; (4) Intermediate osteoblasts (OB5, *NR4A1^high^/NR4A2^high^*) were in the middle stage of the lineage, and had the potential function of regulating osteoclastogenesis and involving in adipogenesis together with OB6; (5) Intermediate osteoblasts (OB6, *ATF3^high^/NAMPT^high^*) were in the middle stage of the lineage. It had immunomodulatory function together with OB1 and had the function of regulating osteoclastogenesis and involving in adipogenesis together with undetermined osteoblasts 2 (OB5); (6) Mature osteoblasts (OB2, *SPP1^high^/BGALP^high^*) appeared at the end of cell differentiation. In our results, the positions of preosteoblasts (OB3) and undetermined osteoblasts (OB5) in the cell lineage have been found in our previously published articles [22], and new functions of undetermined osteoblasts (OB5) have also been discovered [22].

Although we reanalyzed the imputation scRNA-seq data to further reveal the heterogeneity and potential functions of human osteoblasts, an important limitation was that all the cells were derived from a 31-year-old Chinese male subject with osteoarthritis and osteopenia collected from the femoral head [22]. Compared with healthy individuals, this might lead to biases in the identification and especially in the proportion estimation of osteoblast subpopulations. OB1 and OB6 in our results had immunomodulation functions, which may be due to the subject’s osteoarthritis condition, because the inflammatory state may stimulate the formation of such osteoblast subpopulations with special functions. But how disease conditions affect the composition of cell subpopulations is an open and interesting question which needs further research. Despite this potential limitation, our findings provided necessary and valuable insights into the cellular heterogeneity of human osteoblasts *in vivo*, and comprehensively and systematically knowledge in regulation of adipocytes and osteoclasts differentiation and cell-specific mechanisms that may lead to bone metabolism and other related diseases.

## 5. Conclusions

In conclusion, by performed a novel imputation method to resolve dropout events in the scRNA-seq data of freshly isolated human osteoblasts. Three new osteoblast subtypes been identified after the imputation, by analysis the biological processes and signaling pathways in each subtype, these new osteoblast subtypes could involve osteoclast and adipocyte differentiation and immune activation. These findings provided a better understanding about the osteoblast heterogeneity and a further insight into various (pathological) physiological conditions.

## Supplementary Material

Supplementary Material
