# Supplementary material for: Imputation of Human Primary Osteoblast Single Cell RNA-Seq Data Identified Three Novel Osteoblastic Subtypes
Source: Front Biosci (Landmark Ed). Author manuscript; Available in PMC 2024 May 16. (PMC11097352; doi:10.31083/j.fbl2710295)
Supplement: Supplementary Material [file NIHMS1989305-supplement-Supplementary_Material.doc]

Supplementary Material


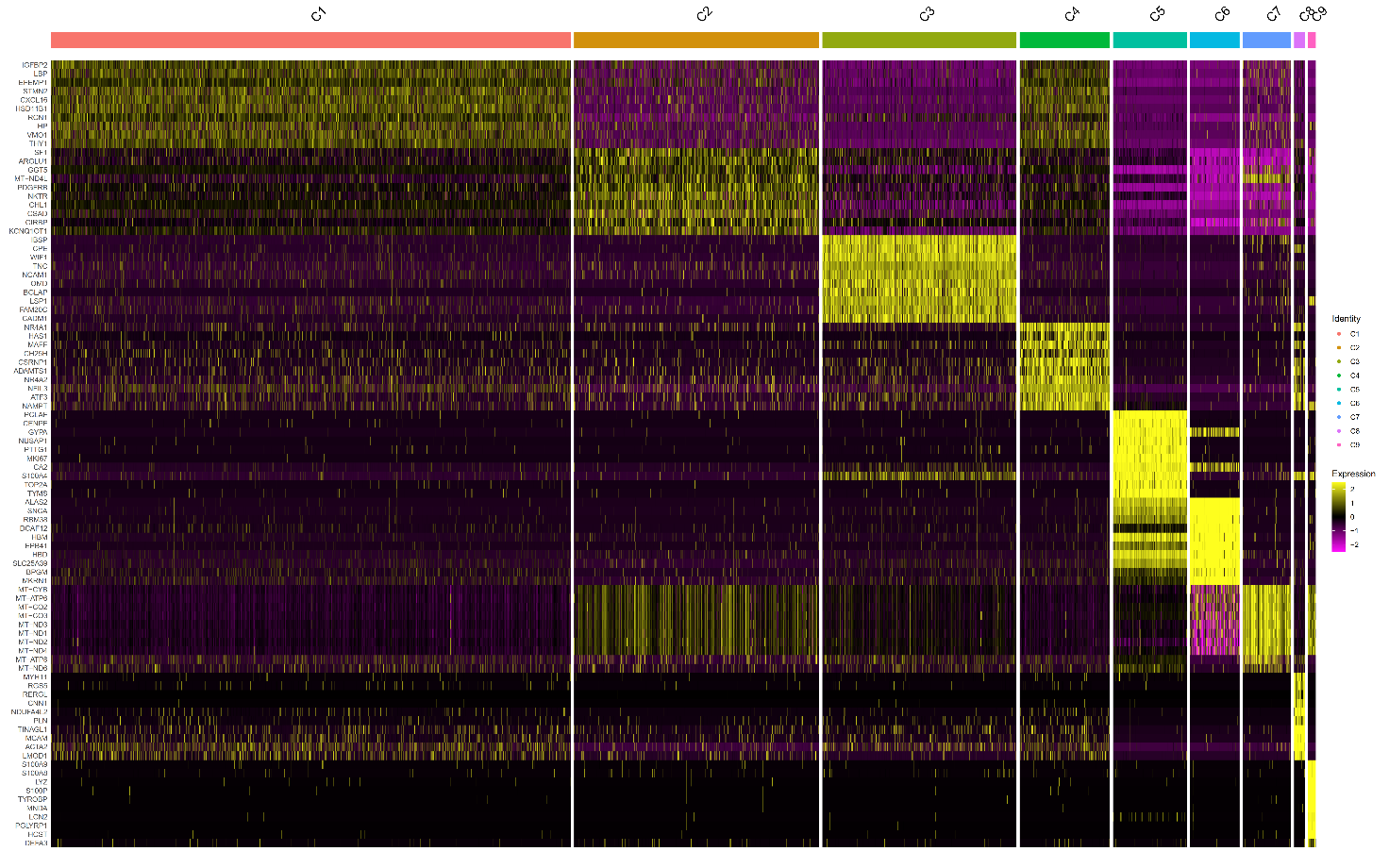


**Supplementary Fig. 1. Heatmap of gene expression profile of isolated cells, based on the relative gene expression level of top 10 most-significant markers for each cluster.** X-axis represents different osteoblast clusters, while y-axis indicates the 10 most-significant markers in each cell cluster.

**
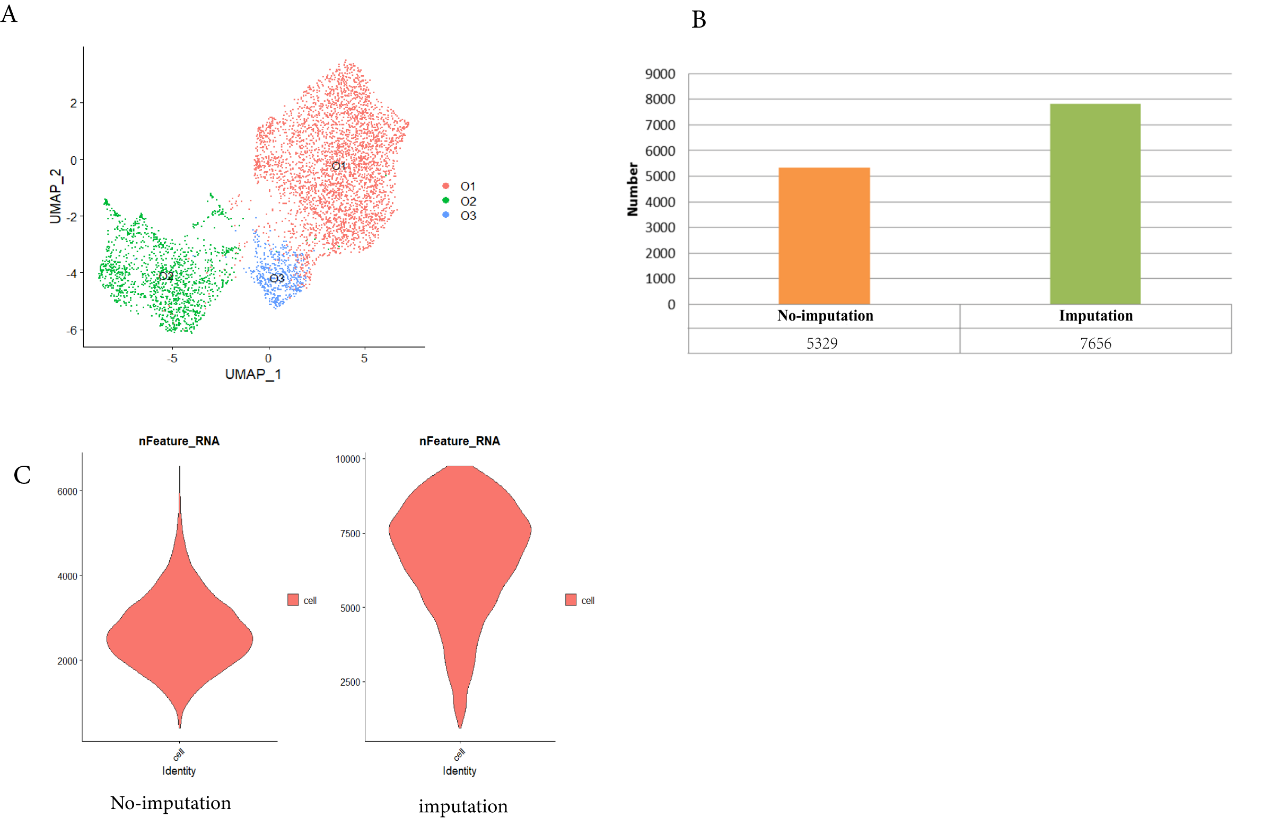
**

**Supplementary Fig. 2.** **Changes of osteoblast transcriptome data before and after imputation.** (A) There were the UMAP reduction of three subtypes of osteoblasts from previous research (O1: preosteoblasts, O2: mature osteoblasts, O3: undetermined osteobalsts (*NR4A1^high^/ NR4A2^high^*)). (B) There were 5329 osteoblast cells without imputation and 7656 cells after imputation. (C) In the raw data without imputation, an average of 2190 genes were detected in each cell, and after the imputation, an average of 6659 genes were detected in each cell.
